# Screening for congenital fetal anomalies in low risk pregnancy: the Kenyatta National Hospital experience

**DOI:** 10.1186/s12884-018-1824-z

**Published:** 2018-05-23

**Authors:** Callen Kwamboka Onyambu, Norah Mukiri Tharamba

**Affiliations:** 1 Department of Diagnostic Imaging and Radiation Medicine, University of Nairobi, Nairobi, Kenya; 2 Mathari referral and teaching Hospital, Ministry of Health, Nairobi, Kenya

**Keywords:** Obstetric, Malformations, Sonography, Low risk

## Abstract

**Background:**

Congenital malformations contribute significantly to the disease burden among children globally. A study conducted in Kenya on understanding the burden of surgical congenital anomalies, highlights the need for Kenyan health systems to go beyond the medical dimensions of illness. This could be achieved by linking knowledge of the severe congenital anomalies (CAs) and their impact of varying disability to the delivery of local health services and public health program planning. Subsequently, early detection of these congenital anomalies is vital and can be achieved through fetal ultrasonography.

Studies have proven that antenatal ultrasound can successfully diagnose fetal abnormalities in many cases and therefore aid in counseling of parents and planning for early intervention.

Although there are studies on screening of congenital anomalies in various populations, very few have been done in the African population and none to the best of our knowledge has been done in Kenya.

**Methods:**

The patients, who underwent routine obstetric ultrasounds, were recruited into the study. The study population comprised patients who were referred from the obstetric clinic, casualty and other clinics within the hospital vicinity. Data of antenatal ultrasounds was statistically analyzed on structured data collection form to determine the prevalence of congenital anomalies.

**Results:**

Fifteen fetal anomalies were diagnosed in 500 women who came for routine ultrasound (3%). The mean age of the mothers was 28.2 years (SD ± 4.5) with an age range from 15 to 44 years. 400 (80%) of the mothers were aged between 27 and 34 years.

The most frequently observed fetal anomalies involved the head (8/ 500; 1.6%). Each of the remaining anomalies affected less than 1% of the fetuses and included anomalies of the spine (2/ 500; 0.4%), pulmonary (2/ 500; 0.4%), renal and urinary tract (2/ 500; 0.4%) and skeletal systems (2/ 500; 0.4%). Majority, 9 of 15 (60%) of the fetuses with anomalies detected on prenatal ultrasound resulted in postnatal mortality within days of delivery.

**Conclusion:**

Congenital anomalies prevalence in our setting compares with those found in other studies. From this study, major birth defects are a major cause of perinatal mortality.

## Background

Congenital anomalies are a significant cause of disability, chronic illness, and childhood death in many countries and affect approximately 1 in 33 infants. They result in an estimated 3.2 million birth defect-related disabilities every year_._ Literature shows that 2–3% of all births are complicated by congenital anomalies and therefore, they are an important cause of perinatal morbidity and mortality accounting for 20–30% of perinatal deaths [1].

This study sought to evaluate the prevalence and spectrum of congenital anomalies on obstetric ultrasound at Kenyatta National Hospital (KNH), being the largest referral hospital in East and Central Africa. Many a number of obstetric scans are performed annually and the exact antenatal prevalence of congenital malformations in our hospital population is unknown.

In a study conducted in South Africa, congenital anomalies were regarded a ‘silent epidemic’. In this study, it was noted that only 26.2% of severe congenital anomalies were diagnosed at birth [2] Therefore, it is necessary to include suitable prenatal, family planning and pediatric facilities into the primary health care delivery system to manage these problems. Initiation of programs to reduce the incidence of congenital anomalies such as Down syndrome and neural tube defects would also be of great importance.

Kenya is a third world country where the social support system is almost non-existent and in a few instances where social structures have been put in place, there is bureaucracy to contend with. As a result, it is a major burden for parents and family to bring up a child with mental and physical handicap. Additionally, we live in a set up where gravid mothers tend to attend antenatal clinics at an advanced gestation if such visits are made. This limits the role of primary prevention of congenital anomalies with folic acid and therefore it leaves ultrasound as the next best alternative to identify these anomalies and plan intervention where possible.

## Methods

### Objective

The main objective of this study was to evaluate the prevalence and spectrum of congenital anomalies on obstetric ultrasound, in unselected population at Kenyatta National Hospital (KNH).

### Study design

This was a descriptive cross-sectional study conducted in the Department of Radiology, Kenyatta National Hospital.

### Setting

Radiology Department of Kenyatta National Hospital.

### Duration

Nine months from March 2015 to November, 2015 with three months of active patient recruitment and subsequent follow up.

### Study population

The study population consisted of gravid patients seeking obstetric services at Kenyatta National Hospital. Study participants were recruited over a period of three months between March 2015 and May 2015 at the radiology department of KNH, with follow up done in the subsequent six months.

The principal investigator and a research assistant were stationed at the radiology unit during operating hours each weekday between 8 am and 4 pm.

All consecutive referrals for obstetric ultrasound were approached and assessed for study eligibility. Eligible patients comprised those who were equal to or greater than10 weeks gestation. This is because studies have shown that some anomalies such as anencephaly can be diagnosed as early as 10 weeks [3]. However it is still appreciated that some anomalies can be missed in this gestational period [4]. Eligible consenting patients were recruited into the study. The obstetric referrals constituted patients from the obstetric clinic, casualty and other clinics within the hospital vicinity. Obstetric ultrasounds were performed using GE LOGIQ P6 PRO or Philips HD11 ultrasound machines, where the fetus was examined systematically as in Table 1 below. Other study variables included age of the patient, gestational age of the patient, parity, previous history of fetus with congenital anomaly and existing maternal illness.

**Table 1:** The anatomical survey at the time of the scan

Head (lateral ventricles, septum pellucidum, cerebellum)	
Spine	
Heart, four chamber view and its position	
Pulmonary system	
Anterior abdominal wall	
Stomach bubble and its position	
Bladder and kidneys	
Skeletal system- extremities	

The purpose of the study was explained to the patient and confidentiality of results assured. Each study participants’ particulars were entered in the machine which included ultrasound registration number, name, age and parity. The obstetric ultrasound examination was carried out using a curvilinear 3.5 to 5-MHz sector transducer. No patient preparation was required except for the few who had first trimester pregnancies. They were asked to fill their urinary bladder by drinking at least 4 to 6 glasses of water, or until they had the urge to void.

The coupling gel was applied on the abdomen and thereafter, a systematic fetal examination was performed as per table 1above. Fetal cardiac activity was evaluated using standard B-mode and M-mode techniques.

The findings were documented and selected images printed on ultrasound thermal paper.

Quality assurance and control procedures were implemented during subject recruitment and data management to ensure standardized data collection and adherence to study protocol. Prior to commencement of fieldwork, the research assistant was trained on study eligibility requirements, recruitment approaches and retention strategies. In addition, an eligibility checklist was provided to guide subject recruitment. All study exclusions and reasons for exclusions were documented.

Statistical analysis was done using an analysis package, statistical package for social sciences (SPSS Version 20). All analysis was based on study objectives. Initial descriptive analysis involved univariate analysis of each demographic characteristic contained in the questionnaire. Sample descriptive statistics such as mean and median were calculated for continuous variables e.g. age and proportions and frequency distributions were computed for categorical variables e.g. gender. The primary outcome was determined by calculating the percentage of all obstetric ultrasounds with evidence of fetal congenital defects. Secondary outcomes were determined by calculating percentages of ultrasounds showing the spectrum of specific congenital defects. The primary outcome of congenital defect was then cross-tabulated against patient characteristics and levels of significance determined.

## Results

**Fig. 1:**
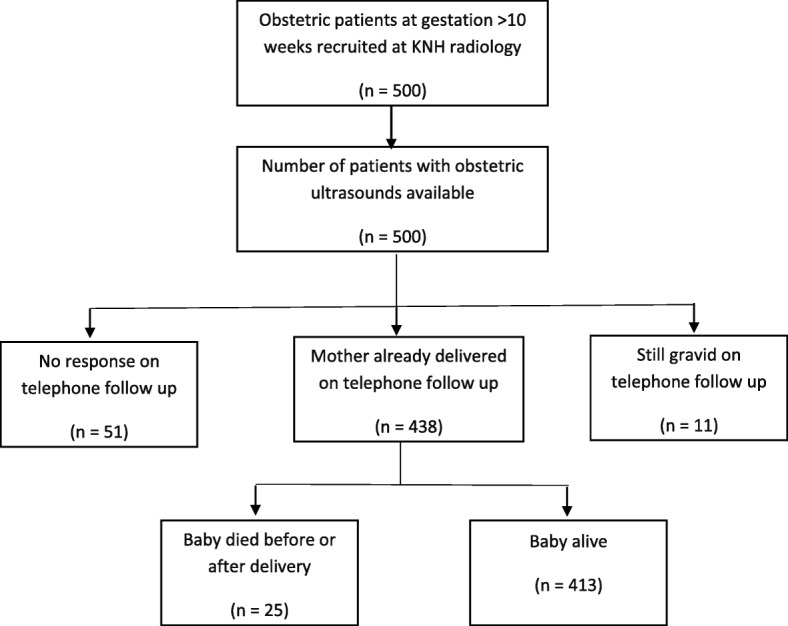
Flowchart of obstetric patient recruitment at KNH radiology unit and follow up

A total of 500 mothers were recruited into the study and had obstetric ultrasounds conducted. Figure 1 below shows the participant flow during the study and prospective follow up rates to determine the outcome of delivery and compare pregnancy outcome to obstetric ultrasound findings. Out of the 500 mothers, 449 (89.8%) mothers were traced during follow up. Of these mothers who were traced, 11 were still gravid, leaving a total of 438 (87.6%) mothers for the final analysis comparing ultrasound findings to the actual neonatal outcome on delivery. Of the babies whose delivery outcome was established on follow up, it was reported that 25 had died before or soon after delivery.

In this population of 500 antenatal mothers offered screening for malformations, a total of 15 fetuses were identified to have congenital anomalies. This corresponds to a prevalence of 3.0% (95% CI 1.5–4.5%).

The mean age of the mothers was 28.2 years (SD ± 4.5) with an age range from 15 to 44 years. Most mothers were aged below 35 years of age, 447 out of 500 (89.4%). Of the 500 mothers seen most had single ton pregnancy with only 2 mothers having multiple pregnancy and neither of them had congenital anomalies.

Fetal congenital anomalies were identified in 15 of the 500 mothers recruited into the study. 400 (80%) of the mothers were aged between 27 and 34 years. 2 mothers were above 35 years of age and there was only one mother aged below 20 years.

Most of the mothers were gravid with either the first 154 (30.8%) or the second 176 (35.2%) pregnancy. There were 64 (12.8%) mothers who had a fourth or higher order pregnancy.Three hundred thirteen mothers were sent for routine obstetric ultrasound. The remaining 187 (37.4%) presented with at least one of the following complaints: pain, decreased fetal movement, per vaginal bleeding, or drainage of liquor.

The median gestation on presentation to hospital for obstetric ultrasound investigation was 32 weeks with an interquartile range from 28 to 34 weeks. Most mothers presented at between 28 and 41 weeks gestation 332 (76%), and 3 (0.6%) mothers presented between 10 and 14 weeks (Table 2). There were no significant differences in the gestational age at which ultrasound investigation was conducted for referrals to KNH and non-referrals (*p* = 0.856).

**Table 2:** Gestation of mothers on presentation for obstetric ultrasound at KNH

	Referral	Non-referral	Total (n)	Percent (%)	*P* value
Gestation in weeks					
Below 14 weeks	0	3	3	0.6	0.856
14–27 weeks	12	92	104	20.8	
28–41 weeks	48	332	380	76	
Not stated	1	12	13	2.6	
Total			500	100.0	

There were 2 mothers who reported that they had previous history of pregnancy with a congenital anomaly and both of them also reported that they were on folate supplementation prior to the current pregnancy.

All anomalies detectable by ultrasound were considered, whether major or minor. Major malformations included lethal or incurable abnormalities and anomalies associated with severe handicap or requiring surgery. Of the 15 fetuses with congenital anomalies, 6 had multiple malformations, which were categorized into the various systems involved.

**Fig. 2:**
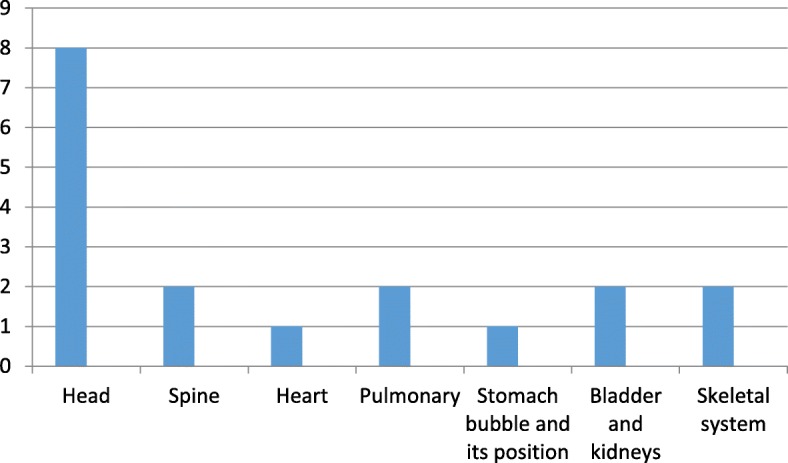
Histogram showing spectrum of congenital anomalies

The most frequently observed fetal anomalies involved the head (8/ 500; 1.6%), Fig. 2, above. Each of the remaining anomalies affected less than 1% of the fetuses and included anomalies of the spine (2/ 500; 0.4%), pulmonary (2/ 500; 0.4%), renal and urinary tract (2/ 500; 0.4%) and skeletal systems (2/ 500; 0.4%). There was a single fetus (0.2%) with congenital anomalies involving the stomach bubble and its position.

The various manifestations of the systems involved are summarized in the Table 3 below with postnatal outcome. Phone follow up of these mothers was done to confirm the prenatal diagnosis in relation to the detected anomalies.

**Table 3:** Systemic involvement of the congenital anomalies and their postnatal outcome

Anomaly		Died	Alive	Termination of pregnancy
Head (*n* = 8/ 15)				
Encephalocele	2/8	✓✓		
Ventriculomegaly	2/8	✓	✓	
Anencephaly	1/8	✓		
Posterior fossa cyst	1/8		✓	
Cystic hygroma (neck)	1/8		✓	
Holoprosencephaly	1/8	✓		
Spine (*n* = 2/ 15)				
Sacrococcygeal mass	1/2		✓	
Scoliosis	1/2	✓		
Heart (*n* = 1/ 15)				
Hydrops fetalis	1/1	✓		
Pulmonary system (*n* = 2/15)				
Pulmonary hypoplasia	2/2	✓✓		
Stomach bubble and its position (*n* = 1/ 15)				
Absent stomach bubble	1/1	✓		
Bladder and kidneys (*n* = 2/ 15)				
Renal agenesis (bilateral)	1/2			✓
Bilateral hydronephrosis? PUJ obstruction	1/2	✓		
Skeletal system (*n* = 2/ 15)				
Femur discrepancy	1/2		✓	
Achondroplasia	1/2	✓		

Four hundred thirty-eight mothers (87.6%) had a telephone follow up done. Out of these mothers who were successfully traced, none reported gross anomalies that were missed on the prenatal ultrasound. However, 4 mothers reported persistent infant chest problems and had frequent hospital visits. These infants could have had possible cardiac anomalies, which is one of the many causes of persistent morbidity involving the chest and for which studies have reported to be easily missed on ultrasound.

Majority, 9 of 15 (60%) of the fetuses with anomalies detected on prenatal ultrasound resulted in postnatal mortality within days of delivery. It was not possible to ascertain the presence of anomalies after delivery for this group of infants in whom early neonatal deaths occurred. Corrective surgery was carried out in the neonate with an absent gastric bubble though mortality occurred due to other complications. One-third, 5 of 15 (33.3%) babies with prenatal diagnosis of anomalies fared well postnatally. Ventriculomegaly was diagnosed in two cases, one of which progressed well into the post neonatal period. However, confirmatory follow up imaging was not carried out in the surviving infant to ascertain the diagnosis.

Femur discrepancy was reported in one prenatal ultrasound, where the right femur measured 6.48 cm corresponding to 33 weeks 3 days gestation while the left was 7.42 cm corresponding to 38 weeks. The mother however reported no noticeable difference in the lower limbs of the baby post-delivery. There was a single pregnancy termination, 1 out of 15, due to multiple anomalies (scoliosis, pulmonary hypoplasia, absent kidneys and bladder), some of which were by themselves fatal.

An isolated case of echogenic intra-cardiac focus at 20 weeks in a 28 year-old primigravida was seen, who on follow up was clinically normal.

There was a significant association between congenital anomalies visualized on ultrasonography and maternal history of a previous pregnancy with congenital anomalies (*p* < 0.001). One out of the two mothers with positive history of fetal congenital anomalies had an anomaly detected on ultrasonography for the index pregnancy. Of the 498 mothers with no history of previous fetal anomalies, 14 (2.8%) had an anomaly detected on ultrasound scan.

**Fig. 3:**
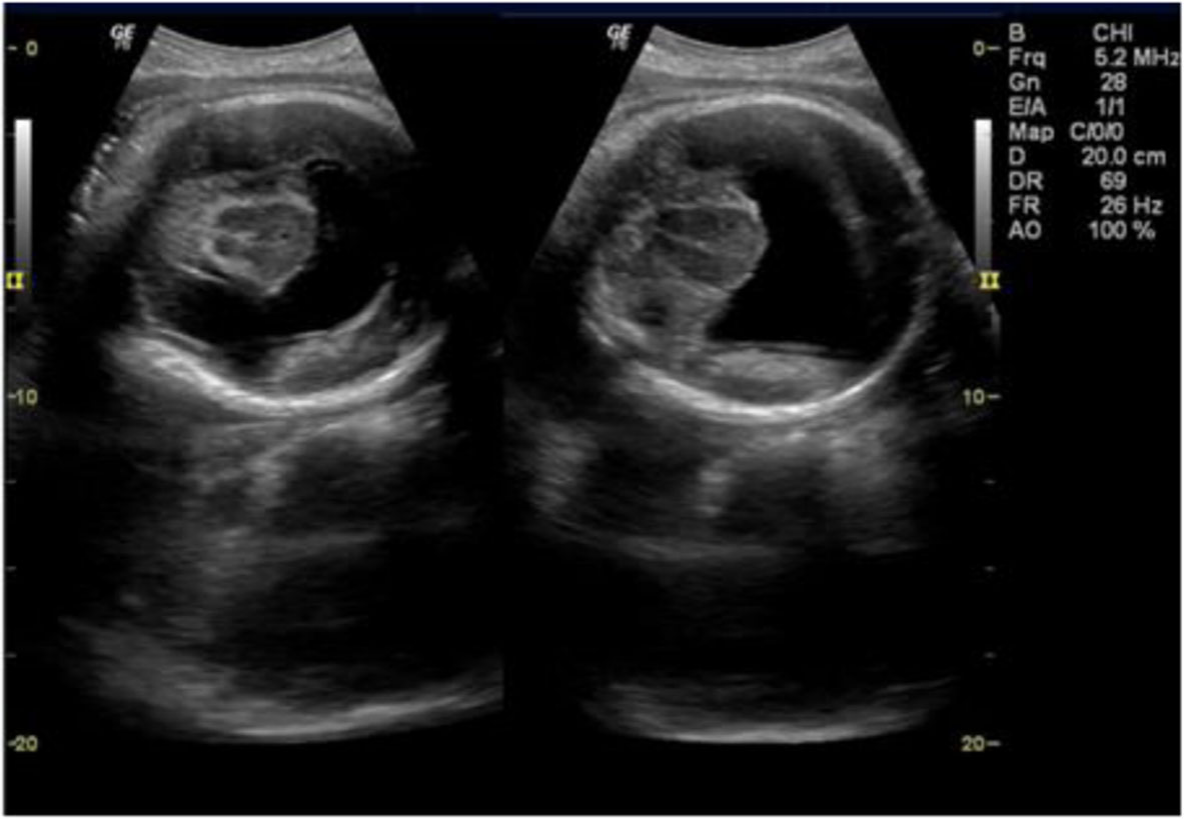
The above images were from a mother in the age range 30–34 years at 38 weeks 5 days gestation who presented with a history of decreased fetal movements. On ultrasound examination, the head showed fused thalami, a monoventricle/holosphere with absent interhemispheric fissure. A diagnosis of alobar holoprosencephaly was made. The other systems were normal. Mortality occurred on the second day post-delivery in the neonatal intensive care unit (NICU)

**Fig. 4:**
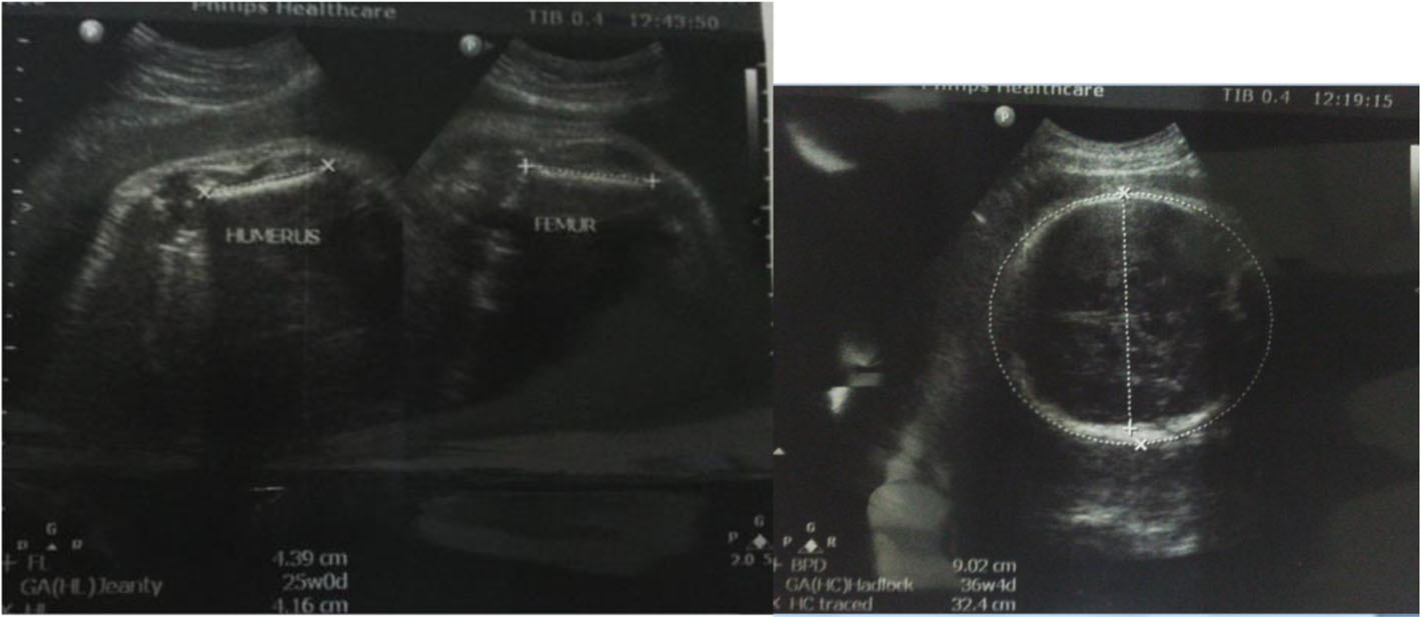
The above images were obtained from a mother age range 35–39 years Gravida 2 who was on follow up due to a suspected anomaly on an earlier scan. Her scan showed a huge disparity between the humeral/femur length and other biometric data. The chest was also relatively small. A diagnosis of probable asphyxiating skeletal dysplasia was made. The findings were confirmed upon delivery. The neonate died shortly after delivery due to respiratory complications

**Fig. 5:**
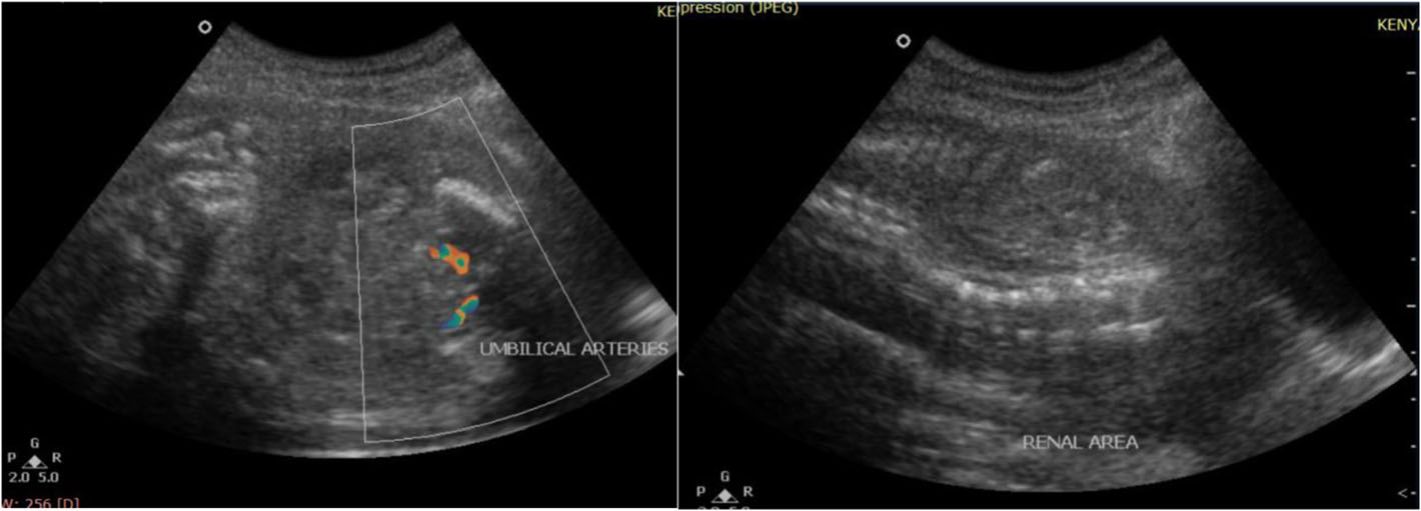
These are images from a mother in the age range 25–29 years Gravida 3 at 31 weeks who presented with a history of fundal height not corresponding to dates. On imaging, both kidneys and the urinary bladder were absent. The thoracic diameter was relatively small likely due to pulmonary hypoplasia and associated oligohydramnios. A diagnosis of bilateral renal agenesis was made. Ultrasonic age corresponded to 25 weeks

**Fig. 6:**
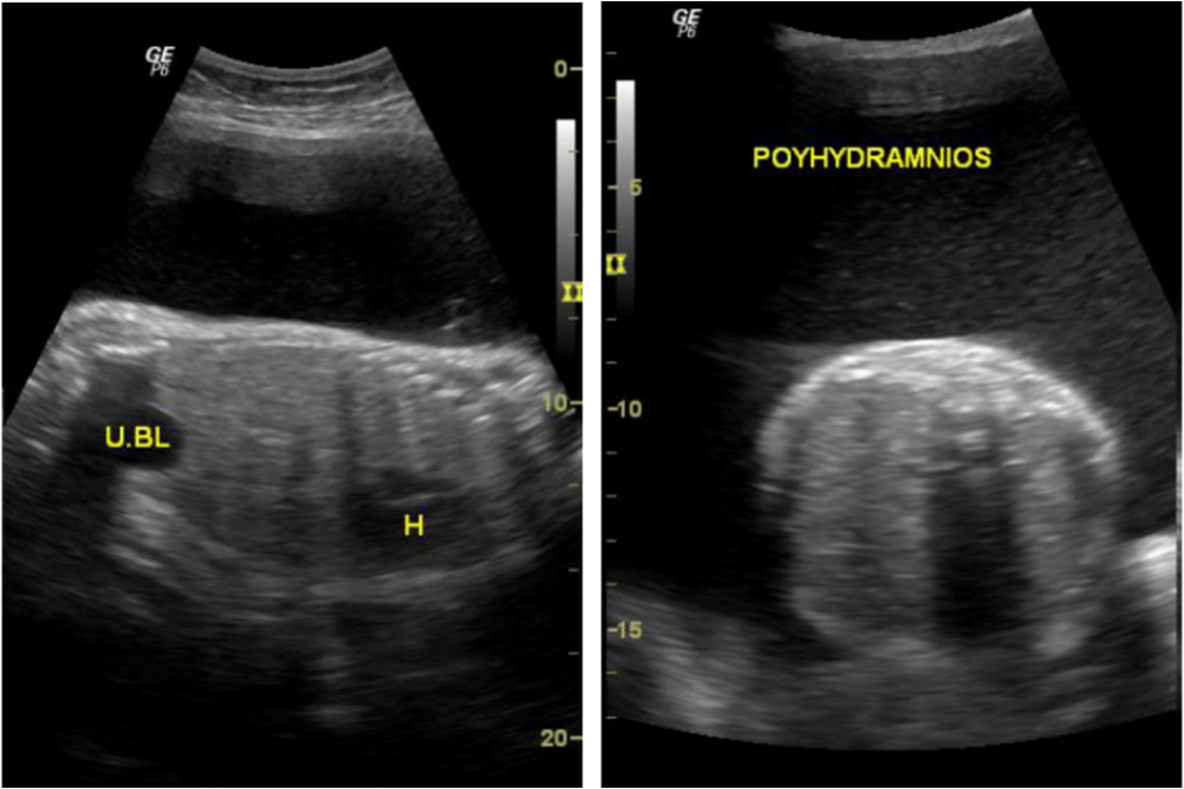
The above images were obtained from a mother age range 30-34 years Gravida 4 on routine sonography to assess fetal wellbeing. She had had previous normal pregnancies and was not on folate supplementation. The scans showed a ventricular atrial diameter of 20.1 mm and a diagnosis of ventriculomegaly was made

**Fig. 7:**
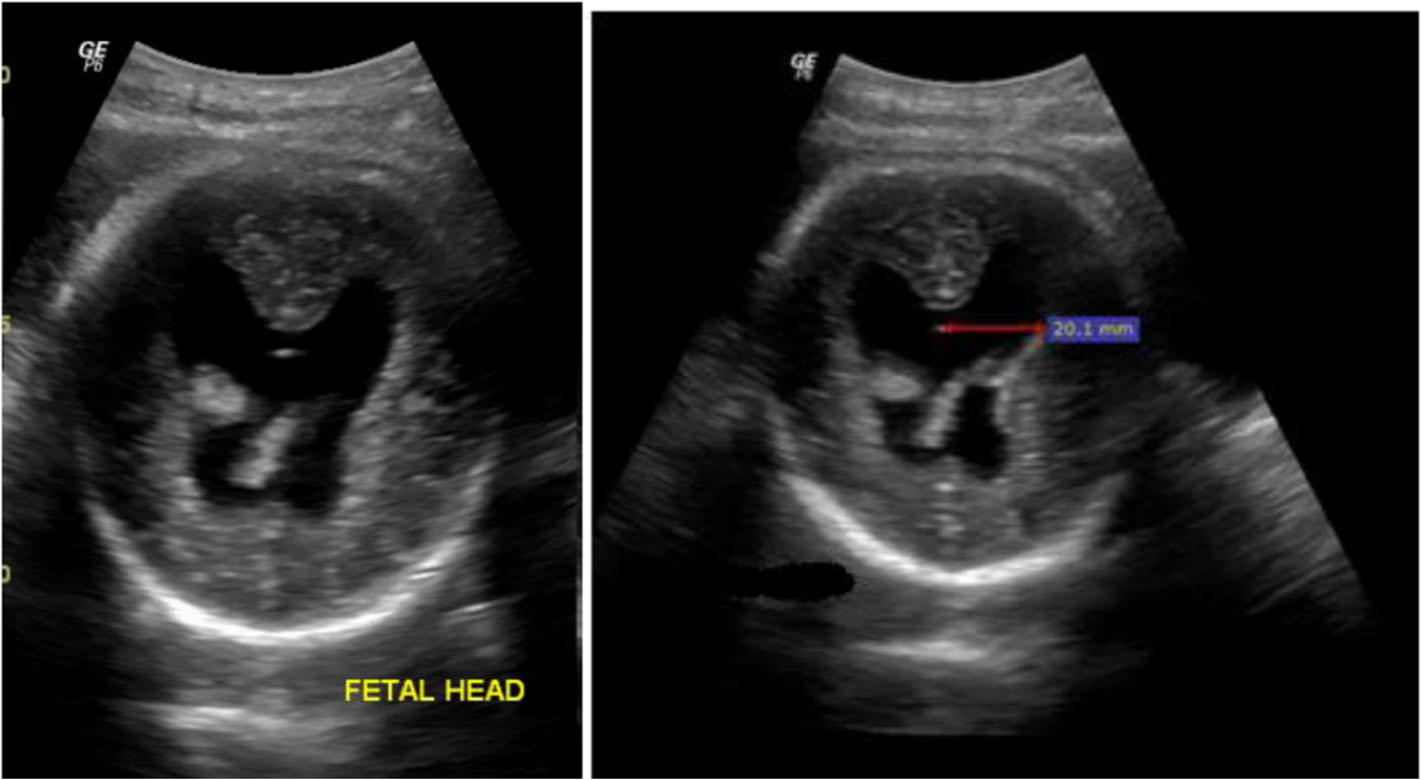
These images are from a mother age range 25–29 years Gravida 3 who presented with a history of abdominal pain and fundal height not corresponding to dates. On examination, the stomach was not visualized during the entire scan period and there was polyhydramnios. A possibility of esophageal atresia was entertained. Upon delivery, the mother reported inability to feed and was informed of a possible “cardiac problem”. Unfortunately, infant demise occurred a few days post-delivery

**Fig. 8:**
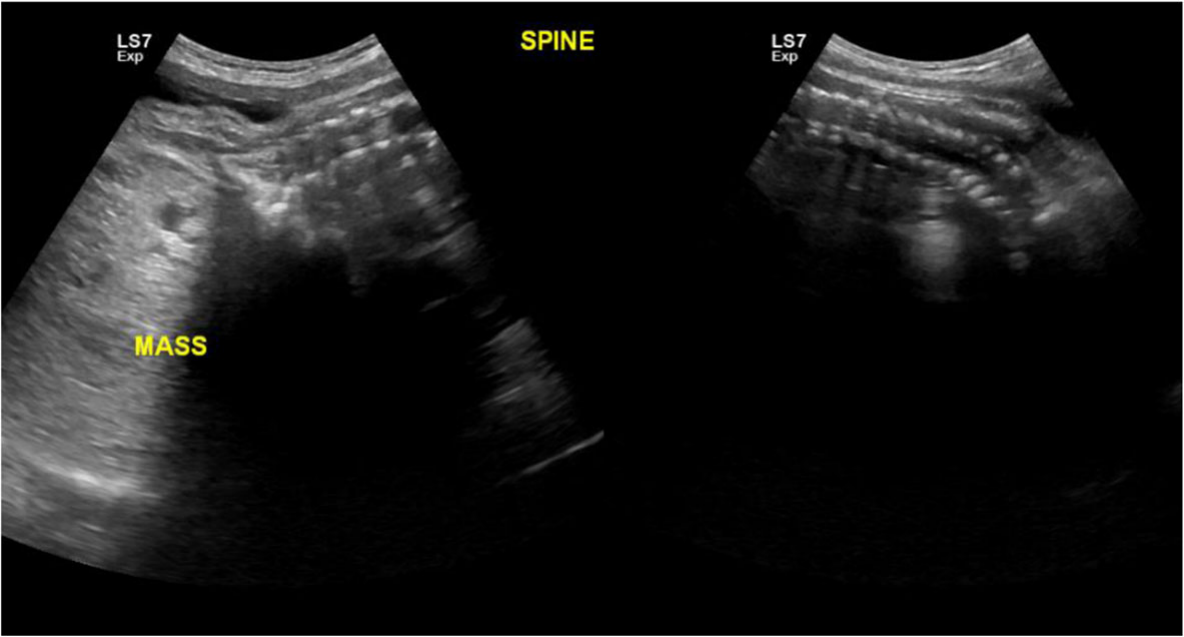
This image was obtained from a mother age range 35–39 years Gravida 3 on a routine obstetric scan. Examination showed an anteriorly placed sacrococcygeal mass likely a teratoma. All the other systems were normal. The diagnosis was confirmed postnatally, with the neonate on follow up at the surgical clinic

Representative images from the study are presented in Figs. 3, 4, 5, 6, 7 and 8 below.

## Discussion

The aim of this study was to evaluate the prevalence and spectrum of congenital anomalies on obstetric ultrasound in Kenyatta National Hospital population. In addition, manifestations of the affected systems and associated maternal risk factors were also assessed.

A total of 500 patients were studied. The age range of our study subjects was expected as it’s the period when child bearing is at its peak. This favorably compares with a KNH based study by Muga R. on congenital malformations among newborns in Kenya [5].

First or second order pregnancies were predominantly documented possibly be due to better family planning knowledge and an increase in contraceptive utilization as shown by a Nairobi study [6]. There was also late clinic attendance, as replicated by an earlier Kenyan study that showed a number of mothers attended antenatal clinic once prior to delivery and the visit was after 28 weeks gestation [7].

Prevalence of congenital anomalies in the sampled population was 3%. This compares to the 2.3% congenital malformations prevalence observed in the RADIUS study [8]. A further study done in Madina Teaching Hospital, Faisalabad on grayscale ultrasound showed a comparable antenatal prevalence of 2.97% [9]. Another similar Saudi Arabian study produced a figure of 2.79% [10].

The central nervous system anomalies were preponderant as was the case in two similar studies, based in Nigeria and South Africa [11, 12]. However, literature suggests that patterns of congenital anomalies may differ between regions [13].

Late gestational age at diagnosis could be attributed to delayed seeking of antenatal care and a lack of uptake of antenatal ultrasounds. Similar findings were found in a Saudi Arabian study on antenatal prevalence of congenital anomalies which showed a median gestational age at diagnosis of 31 weeks [10]. In contrast, the Eurofetus study showed a mean gestational age at diagnosis of 24.2 weeks likely due to the differing health care system and health-seeking behavior in Europe [14].

There was no significant association between maternal age and higher birth order with the prevalence of congenital anomalies. However, studies have demonstrated that these factors pose a significant risk to the occurrence of congenital anomalies [15, 16]. Findings in our study could be due to the fact that majority of the mothers were aged below 35 years and with a lower birth order. This compares to Kenya Demographic Health Survey findings [17].

On the other hand, a previous history of pregnancy with anomaly had a significant association with the occurrence of congenital anomalies. Literature has shown that most anomalies are sporadic or multifactorial, though some developmental anomalies have been found to have an underlying basis on genetics [18].

The study showed that congenital anomalies are a major cause of perinatal mortality. This compares to a Brazilian study on congenital malformations which showed that odds of perinatal death were greater among those with birth defects as compared to newborns without malformations [19].

The possibility of cardiac or lung anomalies that were missed prenatally could not be ruled out in cases where persistent morbidity was reported. These could represent cases of false negative, however this was not confirmed. A Swiss study showed a low detection rate for cardiac anomalies [20] and this may explain our findings. This was also observed in the Eurofetus study that showed a low detection rate for the anomalies of the heart and great vessels [15].

In a Taiwan study, fetal echogenic intracardiac foci were not associated with significant intracardiac or extracardiac anomalies [21]. However, literature shows varied findings with a U.S. study (significance of an echogenic intracardiac focus in fetuses at high and low risk for aneuploidy) that noted there is a raised risk of fetal chromosomal abnormality, most commonly Down syndrome [22, 23].

The limitation of this study was that it was difficult to confirm the anomalies detected at sonography postnatally because of the poor outcome and therefore other confounders as causes of death could not be ruled out.

Another limitation is that follow up was done by phone and therefore this may affect the reliability of the information as reported by the mothers.

Kenyatta National Hospital is a referral hospital; however, most of our study patients were non referrals (87.8%) and therefore constituted a low risk population. This makes our findings fairly accurate.

## Conclusion

Congenital anomalies prevalence in our setting compares with those found in other studies. The study also showed multiple malformations in some fetuses and preponderance of anomalies involving the head. From this study, major birth defects are a major cause of perinatal mortality. Therefore screening for congenital anomalies in obstetric sonography is an important component of primary healthcare for maternal and child health.

## Abbreviations

CA: Congenital anomalies
KNH: Kenyatta National Hospital
NICU: Neonatal Intensive Care Unit
SPSS: Statistical Package for Social Sciences
UON: University of Nairobi
